# *Histoplasma* Peritonitis: An Extremely Rare Complication of Peritoneal Dialysis

**DOI:** 10.1155/2018/8015230

**Published:** 2018-05-10

**Authors:** Asjad Sardar, Bijin Thajudeen, Pradeep V. Kadambi

**Affiliations:** ^1^Division of Nephrology, Department of Medicine, University of Arizona, Tucson, AZ, USA; ^2^Department of Medicine, University of Florida College of Medicine-Jacksonville, Jacksonville, FL, USA

## Abstract

Bacterial peritonitis is a common complication of peritoneal dialysis, but fungal peritonitis is unusual and is mostly due to* Candida* species. Peritonitis due to* Histoplasma capsulatum* is rare and we report one such case. A 63-year-old female presented with progressively worsening abdominal pain, fever, and altered mental status. She had end-stage renal disease and had been on peritoneal dialysis for 4 years. She had abdominal tenderness without rebound or guarding. Laboratory studies and CT of abdomen were significant for leukocytosis and peritoneal membrane thickening, respectively. Peritoneal dialysis fluid study was consistent with peritonitis and culture of the fluid grew* Histoplasma capsulatum*. Treatment recommendations include removal of catheter and initiation of antifungal therapy. With the availability of newer antifungals, medical management without removal of PD catheter is possible, but at the same time if there is no response to treatment within a week, PD catheter should be removed promptly.

## 1. Introduction

*Histoplasma capsulatum* (*H. capsulatum*) is a dimorphic fungus, which is endemic to North America (Central United States; Ohio-Mississippi valley) and Latin America [1–3]. It is associated with exposure to bat caves and avian droppings [4, 5].* H. Capsulatum* peritonitis should be suspected in patients on peritoneal dialysis (PD) from endemic areas who have the potential for exposure. Review of data from United States Renal Data Systems from 1992 to 1997 demonstrated age-adjusted incidence ratio of fungal peritonitis of 9.8 compared with general population and represents 4.5% of total peritonitis episodes in the PD population [6]. The vast majority of these cases are caused by* Candida* species. Mortality secondary to PD associated peritonitis is organism specific: 28% for fungi, 16% for enteric organisms, and 15% for staphylococcal species. The presence of certain additional factors in PD patients increases the risk for fungal peritonitis. Almost all published series have found an association with both recent antibacterial use and episodes of bacterial peritonitis [7–12]. When these series are combined, 65 percent of patients had been exposed to antibiotics within 30 days of the onset of fungal peritonitis, and 48 percent had experienced an episode of bacterial peritonitis within the same time frame. Other risk factors include emergency PD, HIV infection, abdominal surgeries, extraperitoneal fungal infections, and environmental exposures. Details of previously reported cases of* H. capsulatum* are also discussed.

## 2. Case Report

A 63-year-old female who was visitor from Veracruz, Mexico, presented to the emergency room with complaints of progressively worsening abdominal pain and distention for three days. She also had fever and altered mentation. Her past medical history was significant for hypertension, diabetes mellitus, hyperlipidemia, and end-stage renal disease. She had been on PD for four years and denied any recent changes in technique. She had two episodes of peritonitis in the past while in Mexico but was unaware of the details of those episodes. Her surgical history was significant for appendectomy, cholecystectomy, and tubal ligation and she denied any recent abdominal procedure. She denied smoking, alcohol intake, or use of recreational drugs.

**Figure 1:**
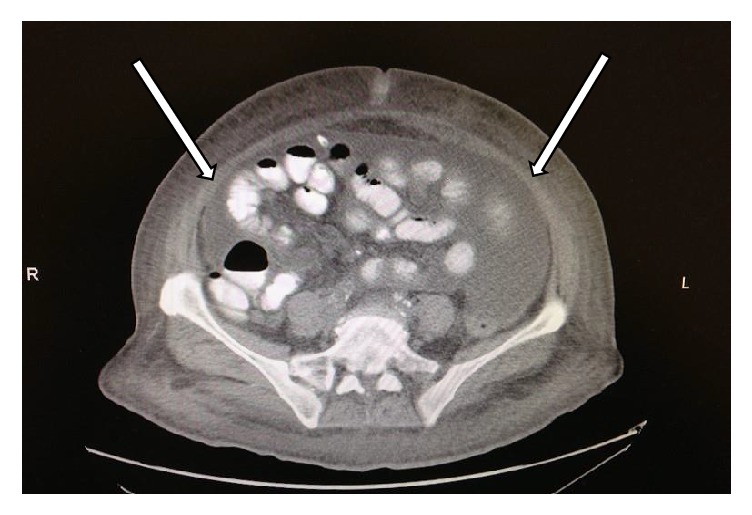
Computed tomography of the abdomen showing peritoneal thickening (arrows), consistent with peritonitis.

On examination, her blood pressure was 172/85 mm of Hg, pulse 88/min, oral temperature 39.5°C (103.1°F), respiratory rate 14/min, and oxygen saturation on room air 94%. She was lethargic and confused. She had abdominal distention and diffuse tenderness without any rebound or guarding. Her PD catheter exit site was clean and dry. Laboratory studies showed white blood cell count of 14.5 × 10^3^/*μ*L with 87.1% granulocytes, hemoglobin of 6.3 g/dL, and hematocrit of 18.4%. Serum chemistries showed sodium of 130 mmol/L, potassium of 2.7 mmol/L, chloride of 90 mmol/L, bicarbonate of 27 mmol/L, blood urine nitrogen of 30 mg/dL, and creatinine of 7.7 mg/dL. Her liver function tests were within normal limits. Computed tomography of abdomen and pelvis without intravenous or oral contrast showed peritoneal thickening consistent with peritonitis, and there was no evidence of perforation or obstruction (Figure 1). PD fluid analysis showed white cell count of 2173 per mm^3^ with 96% neutrophils and red blood cells of <3000 per mm^3^.

Blood and PD fluid cultures were sent, and she was empirically treated for bacterial peritonitis with intraperitoneal cefazolin and ceftazidime. PD fluid gram stain revealed budding yeast; blood and PD fluid cultures did not reveal bacterial growth. Given the high suspicion of fungal peritonitis, immediate removal of the PD catheter was discussed with the patient. She chose not to have the catheter removed, leave to Mexico, and get treated by her own nephrologist. Hence oral fluconazole was started for presumed* Candida* peritonitis. However, six days later, the fungal culture [Mycosel Agar and Brain Heart Infusion Agar] of the PD fluid grew* H. Capsulatum*.

## 3. Discussion

As previously noted, fungal peritonitis is an uncommon cause of peritonitis in PD patients. There are currently no established guidelines for the diagnosis of fungal peritonitis. The International Society of Peritoneal Dialysis (ISPD) recommends repeating PD fluid cell count at day 3 of culture negative peritonitis and employing special culture techniques for isolating uncommon organisms including fungi. [19]. Isolation of* Histoplasma* from culture may take up to 12 weeks but can happen as early as 1-2 weeks. Nonculture techniques available for diagnosis include molecular techniques like polymerase chain reaction, serological tests like complement fixations, and immunodiffusion tests for precipitins [17].

If yeast is seen on initial gram stain, then prompt antifungal treatment should be started as we did in our patient. Although the mainstay of therapy in the past has been Amphotericin B, its toxicity has frequently precluded its use [20]. Experience with the newer imidazoles/triazoles and flucytosine suggests that these agents are well tolerated and efficacious [19].

Regarding the removal of PD catheter, ISPD guidelines recommend prompt removal once fungal infection is identified. Additionally, an appropriate antifungal agent should be continued to 2 weeks after removing the catheter [19]. There are isolated reports of treating fungal peritonitis without removing the catheter, but with varying degrees of success. However, this should be considered an option only if patient's medical condition precludes removal of the catheter [17].

Since peritonitis due to* H. capsulatum* is extremely rare, there are no established guidelines for treatment of this condition. The six reported cases were treated with 6–12-month course of Itraconazole with or without Amphotericin B as noted in Table 1 [13–18].

## 4. Conclusion

Although* H. capsulatum* peritonitis is extremely rare, morbidity and mortality associated with it are high. Diagnosis requires high degree of suspicion based on geography and occupation. In such patients, if yeast is seen on gram stain it would be prudent to remove the PD catheter and consider Itraconazole as first choice of therapy for extended period of time.

## Figures and Tables

**Table 1:** Management of reported cases of PD patients with *Histoplasma* peritonitis.

Cases	Treatment regimen	Treatment	Catheter removed
Case 1 [13]	Oral Itraconazole	12 months	Yes
Case 2 & 3 [14, 15]	Amphotericin B	Unknown	Yes
Case 4 [15, 16]	Fluconazole and Amphotericin B	1 month and 10 days	No
Case 5 [17]	Oral Itraconazole	6 months	Yes
Case 6 [18]	Oral Itraconazole	12 months	Yes
